# Towards autonomous robot-assisted transcatheter heart valve implantation: in vivo teleoperation and phantom validation of AI-guided positioning

**DOI:** 10.3389/frobt.2025.1650228

**Published:** 2025-10-21

**Authors:** Jonas Smits, Pierre Schegg, Loic Wauters, Luc Perard, Corentin Languepin, Davide Recchia, Vera Damerjian Pieters, Stéphane Lopez, Didier Tchetche, Kendra Grubb, Jorgen Hansen, Eric Sejor, Pierre Berthet-Rayne

**Affiliations:** 1 Caranx Medical, Nice, France; 2 Department of Cardiac Surgery, Heart Institute Arnault Tzanck, Saint Laurent du Var, France; 3 Groupe Cardiovasculaire Interventionnel, Clinique Pasteur, Toulouse, France; 4 Division of Cardiothoracic Surgery, Emory University, Atlanta, GA, United States; 5 University Hospital of Nice, Nice, France; 6 3IA côte d’Azur, Nice, France

**Keywords:** TAVI, robot-assisted surgery, autonomy, AI, interventional cardiology, medical robotics, image-guided interventions, medical image processing

## Abstract

Transcatheter Aortic Valve Implantation (TAVI) is a minimally invasive procedure in which a transcatheter heart valve (THV) is implanted within the patient’s diseased native aortic valve. The procedure is increasingly chosen even for intermediate-risk and younger patients, as it combines complication rates comparable to open-heart surgery with the advantage of being far less invasive. Despite its benefits, challenges remain in achieving accurate and repeatable valve positioning, with inaccuracies potentially leading to complications such as THV migration, coronary obstruction, and conduction disturbances (CD). The latter often requires a permanent pacemaker implantation as a costly and life-changing mitigation. Robotic assistance may offer solutions, enhancing precision, standardization, and reducing radiation exposure for clinicians. This article introduces a novel solution for robot-assisted TAVI, addressing the growing need for skilled clinicians and improving procedural outcomes. We present an *in-vivo* animal demonstration of robotic-assisted TAVI, showing feasibility of tele-operative instrument control and THV deployment. This, done at safer distances from radiation sources by a single operator. Furthermore, THV positioning and deployment under supervised autonomy is demonstrated on phantom, and shown to be feasible using both camera- and fluoroscopy-based imaging feedback and AI. Finally, an initial operator study probes performance and potential added value of various technology augmentations with respect to a manual expert operator, indicating equivalent to superior accuracy and repeatability using robotic assistance. It is concluded that robot-assisted TAVI is technically feasible *in-vivo*, and presents a strong case for a clinically meaningful application of level-3 autonomy. These findings support the potential of surgical robotic technology to enhance TAVI accuracy and repeatability, ultimately improving patient outcomes and expanding procedural accessibility.

## Introduction

Transcatheter aortic valve implantation (TAVI) is a major advance in cardiology that allows replacement of the aortic valve without open-heart surgery. It has revolutionized the treatment of severe aortic stenosis, particularly in high-risk patients. TAVI was pioneered by Professor Alain Cribier, a French cardiologist who was looking for alternatives for patients with severe aortic stenosis who were ineligible for traditional surgery. In 2002, after years of research, he performed the first successful TAVI at the Charles-Nicolle Hospital in Rouen, France, marking a new era in heart valve treatment. TAVI was approved in Europe in 2007 and in the US in 2011 for inoperable patients. It was initially developed for patients at high risk of open-heart surgery, often older people with other health conditions. TAVI offered a less invasive option, leading to a faster recovery and fewer complications. As the technology improved and clinical trials showed positive results, the use of TAVI expanded to include patients with intermediate or even low surgical risk, depending on their medical conditions (Leon et al., 2010) (Leon et al., 2016).

With an ageing population in developed countries and broader eligibility criteria, demand for TAVI is expected to grow rapidly, reaching 300,000 procedures per year by 2025, with an annual growth rate of 4%–10%. In 2019, TAVI procedures surpassed surgical valve replacement in the US. This increasing demand could lead to a shortage of trained physicians. Studies also show that centers performing fewer TAVI procedures have higher complication rates, highlighting the need for a simpler, more standardized procedure (Leon et al., 2010) (Leon et al., 2016).

**FIGURE 1:**
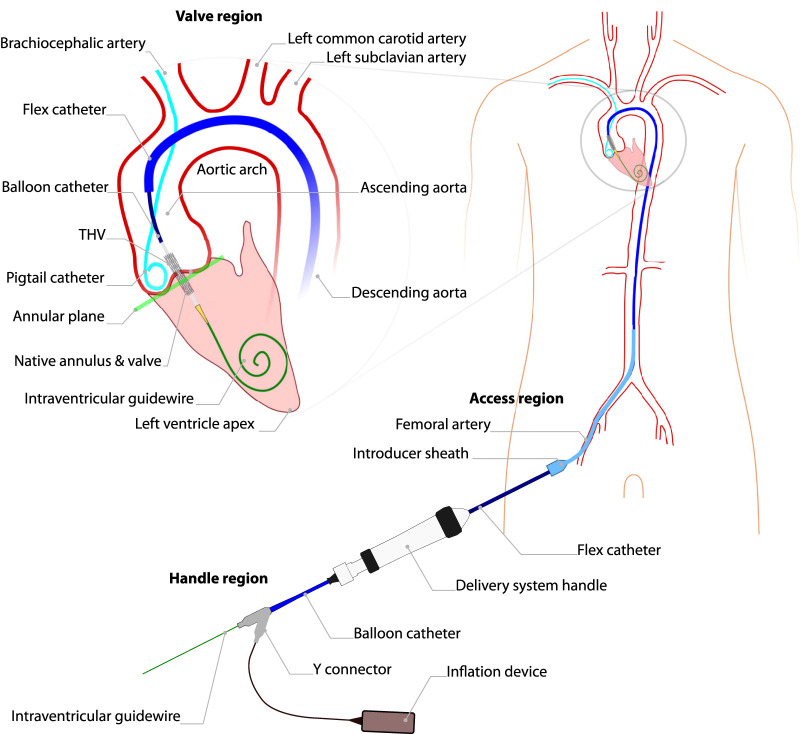
Key anatomy and instruments used during TAVI, using an Edwards Lifesciences SAPIEN3 valve. An outline of the human body emphasizing the vascular and heart anatomy during transfemoral TAVI (right) and a further detailed view of the left ventricle and aortic arch (left). An introducer sheath is present in the right femoral artery, through which an intraventricular guidewire and delivery system catheters are shown inserted. The intraventricular guidewire is positioned within the left ventricle, contacting the left ventricle apex. The flex catheter is positioned along the intraventricular guidewire, with a balloon-expandable TAV implant present on a concentric balloon catheter present within the flex catheter. TAV implantation takes place within the native aortic valve, with the implant being positioned at a pre-planned depth with respect to the planar reference of the native valve cusps, referred to as the annular plane.

TAVI is commonly performed percutaneously, using a minimally invasive approach, with the main instrument pathway passing through an introducer sheath in the groin to access the femoral artery (Figure 1). A guidewire is advanced through the aorta and across the native aortic valve, positioning it in the left ventricle. This wire serves as a track for the delivery catheter, which carries the transcatheter heart valve (THV) to the diseased valve. The prosthetic valve is then deployed, either by inflating a balloon or releasing a self-expanding stent frame that holds the artificial valve leaflets. A pigtail catheter is placed in an annular cusp for contrast injection and as a visual positioning aid, most commonly via secondary radial access. For further procedural details, refer to (Iribarne et al., 2018).

This work focusses on robotic actuation of the valve navigation and delivery phase of the TAVI procedure. Prior to this phase, as depicted in Figure 1, primary access is established, typically in the femoral artery, with a guidewire extending to the left heart ventricle. Secondary access is usually via the radial artery, through which a pigtail catheter is positioned in a valve cusp.

During valve navigation and delivery, two sterile operators and a nurse operate multiple surgical instruments in three areas of interest: access region, handle region and peripheral region. In the access region, located in the groin, one operator maintains the introducer sheath’s position while manipulating the flex catheter over the guidewire through the sheath as shown in Figure 1. In the handle region, the second operator controls the delivery system handle features for catheter control and manipulates the guidewire accessible at the handle’s rear. Additionally, this operator uses a connected inflation device to facilitate THV delivery. Meanwhile, in the peripheral region, a non-sterile nurse manages contrast fluid injections and heart stimulation/rapid pacing with an external pacemaker.

During valve positioning and deployment, the delivery system with the crimped valve at the tip is passed through the aorta to the native valve. Contrast injections are used to visualize and ensure accurate THV positioning with respect to (w.r.t.) the native valve anatomy. Rapid pacing is initiated to prevent THV expulsion into the aorta and the valve is deployed by inflating the balloon at the tip.

A key challenge is to ensure that the THV is deployed to the correct depth relative to the annular plane, as planned before the procedure. Incorrect positioning can lead to paravalvular leakage, valve migration, obstruction of the coronary ostia or conduction disturbances (CD), depending on whether the valve is placed too high or too low (Depboylu et al., 2018) (Van Der Boon et al., 2015) (Athappan et al., 2013).

The incidence of new conduction disturbances (CD) is significant, with a reported incident rates widely ranging from 5% up to 78% (Nuche et al., 2024) and with permanent pacemaker implantation rates commonly reported to be 17% across large patient populations (Abu Rmilah et al., 2022; Siontis et al., 2014). Studies have shown a strong correlation between the depth of THV implantation and the incidence of CD (Almeida et al., 2017; Breitbart et al., 2021; De Torres-Alba et al., 2016; Miyashita et al., 2023; Tarantini et al., 2015). In one study, the pacemaker implantation rate was 13.3% in the correct depth group compared to 21.4% in the deep implantation group (Miyashita et al., 2023). Another study (Breitbart et al., 2021) found that THV positioning was below the recommended zone in 14% of the cases respectively, associating deeper THV placements with a twofold increase in CD rates (52% vs 25%) and a near fourfold increase in pacemaker implantation rates (41% vs 12%). These findings highlight the critical need for accurate valve deployment and the importance of further innovation.

When reviewing TAVI procedures through a technological lens, such innovation may be delivered on several fronts. Interventional imaging overlays, with real-time tracking of anatomy, prosthesis position, and depth error can enhance visual perception of the task space. Repeatable, high-precision actuation with controlled speeds and forces may optimize instrument manipulation, while consolidating the gestures of four hands within a single intuitive operator interface. Combined, the integration of robotic actuation with real-time imaging and guidance presents a unified surgical robotics suite enabling autonomous yet supervised implant positioning, ultimately seeking to improve accuracy and consistency of deployment.

Robotic assistance in structural heart procedures sets out to deliver advancements in medical technology via enhanced precision and improved patient outcomes, while positively impacting control, standardization of skills, ergonomics, and operator radiation exposure. A clear distinction is emphasized: in the same way that interventional cardiology and cardiac surgery are defined by distinct practices and instruments, so too are their respective robotic solutions differentiated.

Robotic assistance in cardiac surgery typically involves direct manipulation of cardiac structures through surgical access (Wei et al., 2022) (Kurnicka et al., 2022) (Kumar, 2019). Invasiveness is able to be reduced as far as a single incision in the chest area in transapical approaches, but still requires open surgical access to the heart to access the left ventricle.

In contrast, transcatheter procedures leverage robotic technology to navigate vascular pathways and deploy devices with high precision via percutaneous access (Cruddas et al., 2021). The heart is accessed by traversing the vascular network using guidewires and catheters, not requiring any chest or pericardial incisions and thus being far less traumatic. Within the domain of transcatheter procedures in cardiology, emphasis has been placed on the navigation challenges, robotically manipulating guidewires and small-bore catheters (<14 Fr) (Robocath, 2017), (Corindus, 2016). Review of using commercial small-bore robotic catheters (Magellan, Hansen Medical) for the navigation and valve crossing phases of TAVI has highlighted reduced vessel wall contact and potentially reduced stroke risk, but did not integrate a THV delivery catheter or review feasibility of robotic THV positioning or deployment (Rippel et al., 2014). In addition to navigation and positioning, preserving haptic feedback while navigating has also been addressed (Woo et al., 2019) (Zhang et al., 2018) and more recently also demonstrated clinically (Sentante, 2024).

This article focuses on robotic assistance in transcatheter procedures for structural heart, applied to TAVI, where large-bore implant delivery catheters (>14Fr) are directly manipulated as per common practice. In the scope of robotizing transcatheter valve implantations, a robotic driver for actuation of commercial delivery catheters has been proposed and characterized without an anatomical simulator (Wang W. et al., 2024). Further related, focus has been placed on robotic manipulators for edge-to-edge repair of the mitral valve (Hausleiter et al., 2023; Zhang et al., 2024). Commercial robotic manipulators aiming at transcatheter valve implantations have also been proposed by PeiJia Medical (WANG D et al., 2022) and Capstan Medical (Capstan Medical, 2015) for aortic and mitral valves respectively. However, at the time of conducting this research and to the best of the author’s knowledge, no technology has been demonstrated to be integrated in a real-world OR environment, or shown feasible *in-vivo* for robot assisted TAVI. Since, initial reports have been made of *in-vivo* demonstrations of robot-assisted transcatheter valve implantations of mitral and tricuspid valves (Wang S. et al., 2024), (Chen et al., 2025), further underlining the contemporary clinical interest and relevance of this research domain and work.

Furthermore, viewed through the lens of autonomy as defined in (Yang et al., 2017), delivery catheter robotic systems often remain limited to tele-operated robotic assistance (level 1). Analogous to the development of transcatheter surgical robotics, advancements are notably focused on navigation through the vasculature. Within interventional cardiology, FDA-cleared transcatheter robotic devices remain limited to level 2 (Lee et al., 2024), while a recent review of the research field has shown *in-vitro* and *ex-vivo* benchtop demonstrations up to level 3 (Robertshaw et al., 2023). Focused on TAVI, the CASCADE European project (Vander Poorten et al., 2016) reported on custom steerable catheters and initial results on level 3 autonomous navigation through the aortic arch on an *in-vitro* simulator. Nevertheless, at the time of writing, focus on delivery catheter manipulation is limited. Feasibility of autonomous valve implant positioning, during which the device positions a large bore THV delivery catheter w.r.t. a tracked anatomy target, has yet to be addressed.

This article seeks to address these gaps by introducing a solution architecture for robot-assisted TAVI, and demonstrating its feasibility *in-vivo*. Furthermore, a framework for level-3 autonomous valve placement is proposed and evaluated using a phantom simulator, applying both camera vision and fluoroscopy for closed-loop control. Finally, an initial operator study is performed to review the added value of various forms of robotic assistance with respect to a manual procedural technique.

## Results

Results are structured as follows. Firstly, robot-assisted TAVI and the novel proposed technology are described. Secondly, *in-vivo* animal verification of the technology on a porcine model is reported. Both tele-operation from a shielded control room as well as robot-assisted valve implantation are demonstrated. Thirdly, *in-vitro* verification of the technology on a silicone phantom model is detailed, during which the added value of both guidance software, robotic assistance, and level-3 autonomy is examined. Finally, feasibility of supervised autonomy is demonstrated using fluoroscopic guidance.

### Robot-assisted transcatheter aortic valve implantation

**FIGURE 2:**
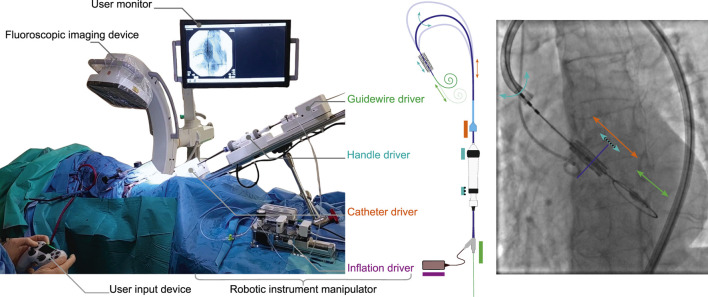
An overview of the technology as deployed *in-vivo* for robot-assisted TAVI. (left) An annotated overview of the system and its main elements as set up on 15th of March 2024, CERIMED Marseille during TAV implantation. (center) The robotic instrument manipulator enabling robotic control over instrument motions for aortic navigation and TAV positioning. A catheter driver and guidewire driver deal with the insertion and retraction of the delivery catheter and intraventricular guidewire respectively. A handle driver enables the delivery catheter flex feature as well as a fine positioning adjustment of the balloon catheter w.r.t. the delivery catheter via actuation of the handle wheels. An inflation driver enables TAV deployment via pressure-monitored balloon inflation. (right) An annotated example of a fluoroscopy image during TAV positioning, highlighting instrument motions in function of each driver of the robotic instrument manipulator.

During TAVI, a plurality of instrument manipulation actions take place throughout the procedure and are generally managed by two operators. Using the proposed technology, manipulation of the flex catheter, handle, and guidewire are done by a robotic instrument manipulator, using a catheter driver, handle driver, and guidewire driver respectively (Figure 2). In the case of a balloon-based valve delivery system, an inflation device is added to enable robotic TAV deployment via balloon expansion. All instrument motion is centralized at a user interface used by a single operator.

Initial development and feasibility review of the technology, prior to *in-vivo* use, was performed *in-vitro* using a custom in-house phantom simulator setup. Simulator realism in terms of anatomical accuracy and internal levels of friction was reviewed by two experienced TAVI operators on-site, in a hands-on qualitative manner. The simulator was considered sufficiently anatomically accurate to represent the positioning and deployment task, while internal levels of friction were considered either comparable or slightly above what is to be expected in a live in-human case, as such representing a useable worst-case scenario for device performance.

All key functionalities were demonstrated tele-operatively, including introducer port fixation, advancing and retracting the delivery catheter, delivery catheter flex, balloon catheter fine adjustment, guidewire advancing and retracting, and balloon inflation (S1). For brevity, a further detailed overview of the technology and its functioning is provided in both methods and supplementary materials.

### *In vivo* robot-assisted navigation in a porcine model

**FIGURE 3:**
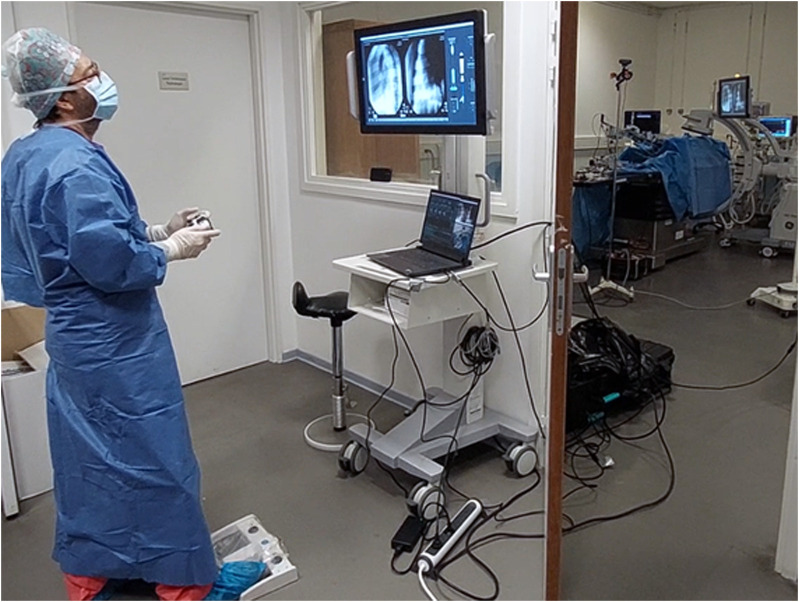
Operator performing robot-assisted TAVI in a teleoperated manner from a shielded observation room, protected from radiation. An expert TAVI operator stands in a shielded observation room, using a wireless handheld interface to control the robotic system during an *in-vivo* animal test on a porcine model. Fluoroscopic imaging is controlled at distance using a wireless pedal. The operator performs the procedure using visual feedback of a monitor display, which shows the live fluoroscopy imaging alongside a visual overview of the robotic system state and robotic instrument manipulations. The right side of the image frame shows the observation room door partially opened to provide a view of the operating room with the robotic system installed on the OR table.

The purpose of these experiments is to review feasibility of interfacing and manipulating instruments safely and effectively in an *in-vivo* setting using the proposed technology. First, with an operator at the bedside, next to the robot. Secondly, with the operator working from a separate radiation-shielded room (Figure 3). Finally, to demonstrate interfacing with the imaging device and collect data for further internal research. These experiments were conducted throughout the period of 15 December 2023 to 16 February 2024 at CERIMED Marseille.

A total of three *in-vivo* experiments were performed, during which both the technology and surgical technique were reviewed and iterated upon. Throughout this campaign, 20 navigation and balloon inflation sequences were executed and logged. In order to perform multiple sequences on a single animal model, no TAV implant was used throughout this campaign.

Teleoperation from a shielded observation room was demonstrated on all three experiments. The operator is maximally protected from radiation while supervising the procedure on the fluoroscopy. The operator is able to navigate the device, position the delivery balloon and TAV implant, and deploy the TAV via balloon inflation using a single remote-controlled user-input device.

For the purpose of this campaign a TAV deployment workflow was emulated via balloon inflation only, excluding TAV implant. A TAV implantation workflow, including implant preparation, was reviewed and tested on the last test day in anticipation of the next experimental goal.

### *In vivo* robot-assisted implantation of a heart valve in a porcine model

The purpose of this experiment is to review feasibility of positioning and delivering a TAV in an *in-vivo* setting using the proposed technology. This experiment was conducted on the 15th of March 2024 at CERIMED Marseille.

Preparation of the animal model was performed as conventional for a two-point access transfemoral TAVI procedure. Delivery system and valve implant preparation was done as per instructions for use by a qualified scrub nurse. Two minor deviations are added, being the placement of two plastic transmission covers on the handle wheels, as well as the addition of an in-line pressure sensor and a 1m length of high-pressure tubing between the delivery system and the inflation device. Introduction of the surgical instruments was done by the operator and scrub nurse. Further details of the preparation phase can be found in methods.

The robotic instrument manipulator is positioned towards the access site, in function of the introducer sheath already present in the femoral artery. The device pose is fixated to match the insertion point and axis of the exposed introducer sheath, optimizing for coaxial instrument insertions. This, to minimize risk of vascular complications as well as instrument insertion loads.

Introduction and docking of the surgical instruments to the robotic device is done by the operator at three areas of interest: 1) The introducer sheath and delivery system catheter are fixated to the catheter driver. 2) The delivery system handle is fixated to the handle driver. 3) The balloon catheter y-connector and intraventricular guidewire are fixated to the guidewire driver. Additionally, the inflation device is fixated to an inflation driver.

The delivery system catheter and guidewire are actuated using friction wheel transmissions, while handle wheels are engaged with geared transmissions. Fixations are done using quick release mechanisms, more specifically thumbscrews and spring-loaded sliders. Both friction-wheel transmissions are engaged using a single tensioning screw, pretensioned manually with a hex key by the operator.

Once all surgical instruments are docked, the operator takes command of the user input device and initiates a calibration procedure to initiate the device for intra-operative use. Handle limits are identified in the direction of no instrument motion, enabling a safe and fast calibration sequence which lasts approximatively 5s. A known positioning range for a known and given type of delivery system is then applied to set the opposing range limits. Once complete, the device is ready for use within known range limits.

Throughout the robot-assisted procedure, the surgical instruments remain accessible for recovery to manual use at all times. Adjustments to the balloon catheter exceeding fine adjustment range remain accessible for the operator manually, as per Instructions for Use (IFU). This is achieved by maintaining backdriveability of the guidewire translation w.r.t. the handle driver.

**FIGURE 4:**
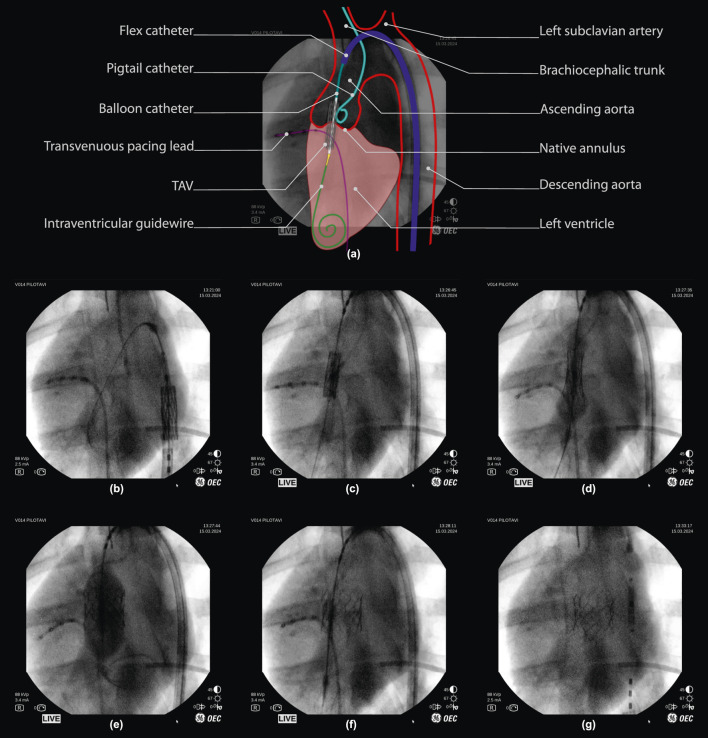
Robot-assisted valve implantation sequence on fluoroscopic imaging, with annotated overview. **(a)** Interpretation of a fluoroscopic image (frame (c), 50% opacity) with an illustrative overlay and annotation of key anatomical features and surgical instruments used during porcine TAV deployment. (below) Fluoroscopy time-sequence: **(b,c)** Tele-operative positioning of the TAV throughout the aortic arch, into the native valve. **(d,e)** final TAV positioning and deployment via balloon inflation. **(f)** balloon deflation, with visual confirmation of initial TAV implantation. **(g)** Tele-operative instrument retraction, with visual confirmation of TAV implantation. *Note that fluoroscopy frames are unprocessed and included as viewed on the operator monitor of the imaging device (OEC One mobile C-Arm, GE Healthcare, Chicago, IL U.S.A.), including timestamps and other on-screen imaging parameters.

The operator tele-operatively navigates and positions the valve implant using the robotic instrument manipulator to control all instrument motion, using the fluoroscopy as visual feedback (Figures 4a,b). When not in motion, the delivery catheter is reliably maintained in a stable position for visual review. It should be noted that, when performed manually, this is done by two operators. Robotically, this can be achieved by a single operator who has centralized command over all relevant instrument motion.

Once in position, the operator tele-operatively deploys the valve implant (Figure 4c). The inflation driver actuates the inflation device at a fixed flowrate (approx. 5mL/sec) to inflate the nominal volume as planned for the chosen TAV (S2). This, while remaining below a pre-determined safe pressure limit (4 bar) using an in-line pressure sensor. The operator retains full control over the inflation via a dead-man switch and is able to pause and continue inflation at will (Figure 4c). Valve deployment is obtained at either full injection of the planned balloon volume or after reaching a pre-defined pressure limit (Figure 4d). Once deployed, the inflation device is used to perform a rapid retraction of the plunger to full retraction, in order to deflate the balloon (Figure 4e). Throughout deployment, the operator remains in full control of all instrument motions to perform any adjustments if required.

Following deployment and balloon deflation, the operator tele-operatively retracts the instruments from the valve region. Stable TAV implant deployment was validated visually on fluoroscopy by an expert TAVI operator directly following deployment, and confirmed once more 5 min after (Figure 4f).

Once valve function is confirmed, instruments are undocked and removed manually from the femoral access region. The procedure is then finalized with access site closure steps as conventional for a three-point access transfemoral TAVI procedure. The overall positioning and deployment sequence was performed in under 1 minute. This is on par with TAVI performed by two operators as per commonplace procedure.

Overall, results show that robot-assisted TAV positioning and deployment is feasible in an *in-vivo* setting on pig model using the developed technology.

### *In vitro* verification of the technology on a phantom simulator model

As a final evaluation, a feasibility review of positioning accuracy and precision using three types of technological augmentations was conducted with three experienced TAVI operators (>3years experience, annualized volume >50 per year) on a phantom simulator setup. During each experiment, an operator is asked to position the delivery catheter at a pre-planned implantation depth w.r.t. an annular reference plane, after which the remaining distance to the intended target is reviewed (S3). Camera vision is used as a radiation-free alternative to fluoroscopy feedback and is displayed on a monitor display.

4 cases were compared: manual instrument manipulation without visual augmentation, manual with visual augmentation, robotic teleoperation with visual augmentation and robotic with automated positioning and visual augmentation. These represent an escalating augmentation of a perception-cognition-action loop: visual perception is augmented using feature tracking and real-time overlay of key features in the operating field of view. This is followed by action augmentation through addition of robotic teleoperation of instruments. Finally, the full perception-cognition-action loop is enabled by implementing closed-loop position control, activated under supervision of the TAVI operator. The test protocol and data-postprocessing are further detailed in methods.

Positioning performance is determined using the distance error of the instrument as indicated by the operator. A positioning error sample is taken once final position is announced by the operator, and is computed as the 3 s average value of the orthogonal distance w.r.t. the implantation depth target. The latter being a fixed depth target w.r.t. the native annular plane and dynamically tracked and visualized for the operator. Accuracy is reviewed using the absolute distance error. The Interquartile range (IQR) and complete data range is used to review precision.

**FIGURE 5:**
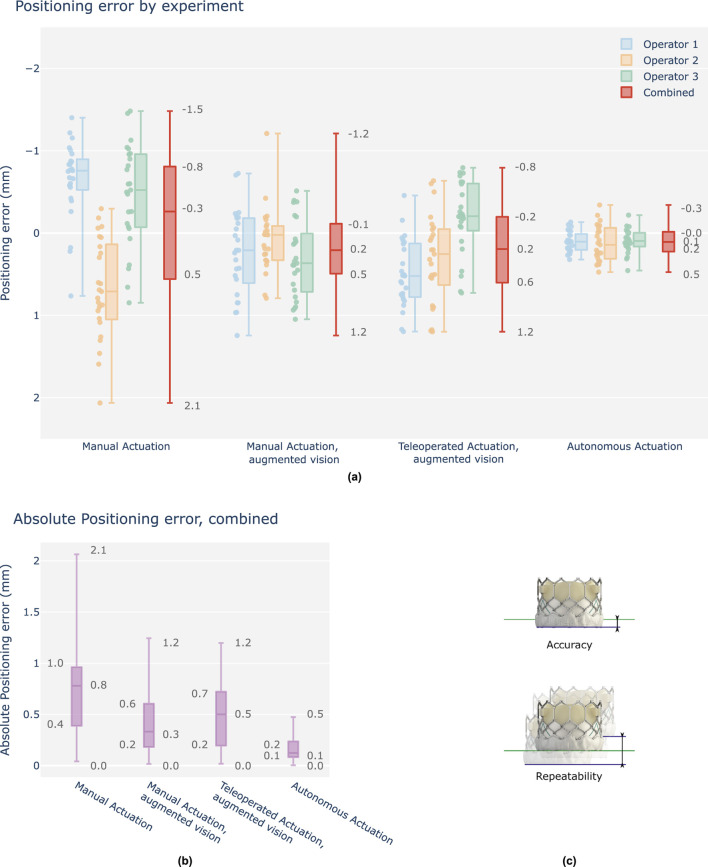
Overview graph of operator positioning performance, final position errors w.r.t. the target depth, signed values in mm. **(a)** Results are grouped per test case and shown as boxplots of which the median, interquartile range (box edges), and highest and lowest sampled values (whiskers) are shown. Each case contains three individual operator results (25 samples), followed by a fourth boxplot representing the combined result across all operators (75 samples). Y-axis is reversed to indicate a positive depth downwards (TAV deeper into the left ventricle), as is commonplace in clinical literature. **(b)** Combined results, shown as absolute values for evaluation of positioning accuracy. **(c)** Accuracy is determined as the absolute positioning error w.r.t. the position target. Repeatability, herein also defined as precision, is determined as the total range variation of the unsigned values.

Figure 5a shows a relative comparison between cases. Individual operator results are shown, as well as the resulting combined outcome per case. Figure 5b shows the combined results as absolute error values for a visual representation of overall accuracy per case. Table 1a summarizes all combined values for relative comparison. For interpretability, camera pixel measurements are approximated in mm’s using a constant conversion factor of 0.16 pix/mm, locally calibrated in the region of interest of the camera frame. The following is observed. Absolute median values for all tested technological augmentations demonstrate improved positioning accuracy compared to the manual reference case. Both IQR values and complete data range values for all tested technological augmentations remain below the manual case indicating improved precision. Complete range of the autonomous solution shows to be approximately 2.7 mm (17 pix) below the manual actuation cases.

**TABLE 1:** Overview table of operator positioning performance, final distance errors, combined. (a) Signed and absolute values in mm. (b) Statistical analysis for each case pair, using t-tests and F-tests.

(a) Case	Accuracy absolute positioning error [mm]			Repeatability signed positioning error [mm]		
	Median	IQR	Range	Median	IQR	Range
Manual	0.75	0.57	2.02	−0.26	1.32	3.54
Manual, augmented vision	0.33	0.41	1.23	0.21	0.6	2.45
Teleoperated, augmented vision	0.50	0.52	1.18	0.19	0.8	1.99
Autonomous	0.12	0.15	0.47	0.11	0.23	0.81

(b) Setup pair	Accuracy t-tests, mean absolute error			Repeatability F-tests, variance unsigned error		
	t	p-value	(p < 0.05)	F	p-value	(p < 0.05)
Manual vs. Manual, augmented vision	5.05	3.0e-06	Yes	3.07	2.8e-06	Yes
Manual vs. Teleoperated, augmented vision	4.07	0.00012	Yes	2.27	0.00051	Yes
Manual vs. Autonomous	11.15	1.6e-17	Yes	24.43	2.2e-16	Yes
Manual, augmented vision vs. Teleoperated	−1.28	0.2	No	1.35	0.2	No
Manual, augmented vision vs. Autonomous	5.81	1.5e-07	Yes	7.97	2.2e-16	Yes
Teleoperated vs. Autonomous	8.35	2.8e-12	Yes	10.75	2.2e-16	Yes

Table 1b summarizes the statistical analysis for each setup pair for both accuracy and precision, applying t- and F-tests respectively. All tested technological augmentations demonstrate significant improvements w.r.t. the manual base case. No significant improvements are observed between manual operation and teleoperation, when both performed with the aid of visual augmentation. Autonomous actuation is observed to be significantly more accurate and precise w.r.t. all other test cases.

Results show, at minimum, equivalent accuracy for each tested solution compared to the manual reference case. Additionally, initial data suggests significant improvements, with up to 2x in accuracy and 4x in precision via the use of autonomous actuation relative to manual positioning. Furthermore, while inter-operator variability decreases with the addition of visual augmentation, a relative improvement of up to 2x in both accuracy and precision remains between autonomy and any tested solution.

Overall, results support the claim of offering increased end-point accuracy and precision with robotic TAVI and level 3 autonomy for THV positioning.

### Fluoroscopy-based autonomous balloon positioning

The purpose of this experiment is to review feasibility of performing automated positioning of a TAV delivery catheter in a real-world setting using the proposed technology in combination with a third-party fluoroscopy imaging system.

Initial setup in hybrid room and data gathering was conducted on 26 October 2023 at Hôpital Privé Arnault Tzanck (*Saint Laurent du Var, France*). The robotic manipulator and phantom simulator were installed and tested in a hybrid room. Six navigation experiments were conducted to assess robot functionality and gather fluoroscopy data on the phantom. Two experiments were remotely controlled by a cardiologist to minimize radiation exposure. Fluoroscopy data from these experiments were combined with data collected at Nice University Hospital and CERIMED under similar conditions. The annotated dataset was used to train a neural network, further detailed in methods. This neural network was subsequently incorporated into the computer vision component of the autonomous positioning algorithm.

**FIGURE 6:**
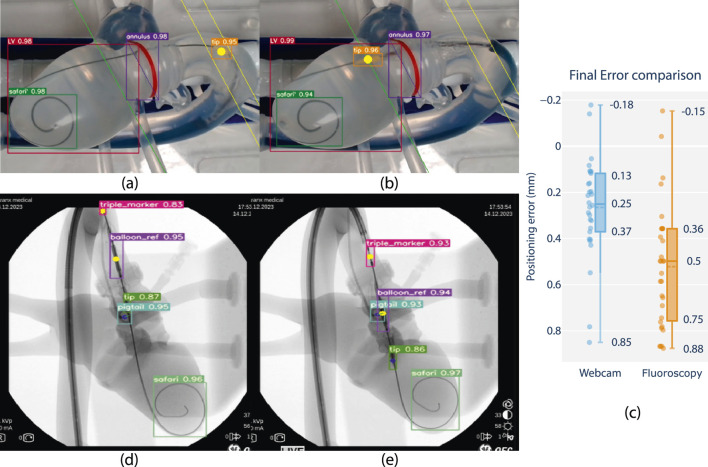
Feasibility review of closed-loop positioning using camera and fluoroscopy on phantom, comparing closed loop positioning errors. **(a)** Starting position and **(b)** final position of camera-based positioning. The balloon tip marker is pre-positioned in the ascending aorta, after which it is moved to match a planned insertion depth at a distance offset a tracked annular reference (green line). **(d)** Starting position and **(e)** final position of fluoroscopy-based positioning. The balloon reference marker is pre-positioned in the ascending aorta, after which it is positioned to match the same insertion depth as the center of a tracked pigtail reference. **(c)** Boxplot comparison with closed-loop positioning error performance using fluoroscopy w.r.t. the camera vision-based lab setup (in mm).*Note that fluoroscopy frames are overlayed with feature tracking and included as screen captured live on a laptop monitor display during the experiment. Raw frames are grabbed from a mobile C-arm (OEC One mobile C-Arm, GE Healthcare, Chicago, IL U.S.A.), including timestamps and other on-screen imaging parameters, and resized to a rectangular aspect ratio for visualization.

Integration and algorithm deployment was conducted on 14th of December 2023 at CERIMED Marseille. Feasibility of closed loop positioning of the delivery balloon w.r.t. a tracked anatomical target was demonstrated using fluoroscopy in a real-world end-use environment (S4). A positioning experiment was conducted 30 times with no system failure. Final positioning error is determined as 0.5 mm median error with a min-max range of 1.03 mm. When repeating the experiment using the camera vision testbench and contrasting the results, positioning performance in terms of accuracy and repeatability was demonstrated to be equivalent across imaging methods in static phantom conditions Figure 6. These observations support the claim of extending experimental observations using camera vision to a fluoroscopy setting, as well as the relevance of continued research in a non-fluoroscopy environment.

Overall, results show that feature tracking and closed-loop positioning on fluoroscopy is feasible using the developed technology. Level-3 autonomy is demonstrated on phantom using fluoroscopy in a real-world end-use environment.

## Discussion

The technology as currently reported is a first-generation prototype and demonstrates feasibility of robotic TAVI while validating device concept, architecture, and theory of operation up to an acute *in-vivo* animal setting. Ongoing developments are focused on a next-generation device intended for investigational clinical use, during which in-human feasibility and safety are addressed. This includes re-engineering hardware and software in accordance with medical device design controls and applicable harmonized standards. At the time of article revision a clinical grade device has been developed, a first FDA-clearance for an AI-based guidance software for in-human use has been obtained (July 2025, K243884), and preparations for clinical trials are ongoing.

Tele-operative feasibility has been demonstrated *in-vitro* and *in-vivo*, where a single operator controls instrument motions using a remote-controlled interface. When applied to TAVI, the tele-operative paradigm stands to offer several benefits. Increasing the operating distance from the radiation source is a core principle for radiation safety, with exposure being inversely proportional to the square of the distance from the source. In particular the first operator (Goel et al., 2022), holding the introducer sheath and advancing the delivery catheter in close quarters with the C-arm, is likely to strongly benefit from being able to work at increased distance. Enabling a position change withing the OR from 0.5m to 1.5m here would render a 9-fold reduction in radiation exposure during TAV deployment. This technology provides operators with the choice to position themselves at safer distances from radiation sources, up to the point of performing the procedure from a fully shielded observation room, contributing to operator safety and wellbeing. Furthermore, using robotics, all instrument gestures can be centered on a single input device, enabling a single human operator to perform the tasks of what otherwise requires two pairs of hands. In view of increased patient populations and a growing shortage of qualified operators, a safe and effective single-operator TAVI stands to enable greater access to care.

Moving beyond the tele-operative paradigm, this technology paves the way for higher levels of autonomy, during which TAV placement can be performed in an automated manner under expert supervision. This is currently envisioned using fluoroscopy feedback as positioning input. This work demonstrates feasibility of doing so on a phantom simulator using both camera vision and fluoroscopy feedback, using live AI inference to track features in real-time. Stable controller convergence is reliably demonstrated using a critically damped controller response, and deemed acceptable when reviewed by expert TAVI operators. Integration in an end-use environment was demonstrated. Future work is focused on demonstrating feasibility of such systems to *in-vivo* imaging.

An initial study was conducted to explore the added value in terms of TAV positioning performance, reviewing technological augmentations of the TAVI procedure relative to a baseline where a human expert operator performs the same task manually, as commonly practiced. Pre-deployment implantation depth error w.r.t. pre-operative planning is used as performance metric. The addition of Level 3 autonomy significantly improves both accuracy and precision on TAV positioning when compared to manual, visually augmented, or tele-operative cases. Relative to the manual baseline, an increase in accuracy and precision of up to 4x is demonstrated. Overall, results indicate that TAVI poses a strong candidate for clinically-relevant supervised Level 3 autonomy, seeking to deliver gold-standard accuracy in a highly consistent manner. Further research is warranted, and would benefit from expanding the experiment to an *in-vivo* setting as well as differentiating on operator experience. As previously noted, with increasing patient populations and a shortage of qualified operators, safe and effective Level 3 autonomy features could expand access to care. These features would enable low-volume and non-expert operators to achieve expert-level precision in valve positioning and deployment, thereby making the procedure more accessible while maintaining a high standard of care.

The current technology demonstrates robot-assisted TAVI with a balloon-inflatable delivery system. TAVI is performed using a variety of valves and delivery systems. It is readily understood that the solution architecture and theory of operation remains generally applicable to other TAVI delivery systems, granted a different interfacing design for each delivery system handle. A generic re-usable robotic base is envisioned, paired with dedicated sterile interfacing cassettes for specific delivery systems. Furthermore, while the current solution architecture is focused on TAVI, it is readily understood that the principles of operation are transferable to other transcatheter valve implant platforms such as mitral or tricuspid. At the time of writing, a similar architecture has been demonstrated for mitral valves (Capstan Medical, 2015).

This work is oriented at demonstrating feasibility of a solution architecture for robot-assisted TAVI in both phantom and acute *in-vivo* settings. Several areas of improvement remain. The current results were obtained using a mobile C-arm with imaging quality below the current state-of-the art of modern hybrid ORs. While sufficient for quantifying feature positions on phantom, a higher quality imaging setup would strongly benefit quantified research on TAV positioning performance *in-vivo*.

Furthermore, the study was performed without a dedicated contrast injection device, relying on manual syringe injections to inject contrast agent in the aortic root which are limited in burst flow rate. This limits the implant positioning target to the visual reference of the pigtail catheter in the Non-Coronary Cusp only. The addition of a contrast injection device, as per common practice TAVI, would enable improved visualization of the target anatomy throughout deployment. Another point of interest would be capturing and quantifying instrument interaction forces as they occur throughout *in-vivo* use. Quantified understanding of anatomical interaction forces using integrated force sensors could support development of more intelligent or adaptive safety functions. No meaningful impact on intraoperative task time was observed during the positioning and deployment phase in both *in-vitro* and *in-vivo* settings. Nevertheless, a full workflow evaluation including device set-up in a routine clinical setting would be of interest in order to evaluate impact on OR time.

With regards to autonomous positioning, the current results are demonstrated on a phantom simulator under quasi-static pressure conditions, at qualitatively-reviewed worst-case frictional forces. Deformation and elastic behavior remains limited to those induced by the instrument interaction forces and any variance in pressure or flow. While motion variance of the implantation target remains relatively small during rapid pacing, the current quasi-static target does present a simplification. Furthermore, the ability to track dynamic heart-cycle motion remains to be characterized. A testbench able to simulate anatomical motion of the heart cycle during normal rhythm as well as during rapid pacing would be of interest.

Overall, this study demonstrates that robot-assisted TAVI is technically feasible *in-vivo* and presents a strong case for a clinically meaningful application of level-3 autonomy. These findings support the potential of surgical robotic technology to enhance TAVI accuracy and repeatability, ultimately improving patient outcomes and expanding procedural accessibility.

## Materials and methods

### Study design and objectives

The main objective of this study was to review feasibility of robotic teleoperative positioning and deployment of an aortic valve implant. This, using a novel robotic platform which was developed for this purpose. More specifically, a robotic device which can be teleoperated, and which is capable of manipulating all surgical instruments conventionally used during the implant positioning and deployment phase of TAVI.

The study design and its associated methods are structured as follows. Firstly, the proposed system architecture of the developed technology and its core functions are reviewed on a phantom simulator. Secondly, feasibility of integrating and using the technology in an *in-vivo* setting is reviewed, during which all instrument motion functionalities as well as the *in-vivo* model suitability itself are reviewed on three animal models. Thirdly, feasibility of performing a complete robotic surgical workflow for positioning and delivering a THV implant is reviewed *in-vivo* on a single animal model. Fourthly, a review of positioning accuracy and repeatability using three types of technological augmentations with respect to a manual technique was conducted on phantom, with an experienced TAVI operator. This involved the addition of feature tracking CNN on camera vision and a closed loop controller enabling automated catheter positioning. Fifthly, the feasibility of performing closed loop autonomous control by interfacing with third party fluoroscopy imaging is evaluated on phantom, involving the additional development of a dedicated feature tracking CNN.

### Robotic system

A feature need analysis was carried out and led to the minimum viable system architecture for balloon-expanding TAVI. Functions were clustered into four driver modules, each handling actuation of a distinct interaction area with surgical instruments as described in the results section. For brevity, technical details about the driver implementations, as well as the input controller and Graphical User Interface are provided in S5.

### Test benches

#### Phantom testbench

A testbench was built around a custom-made deformable silicone phantom (Pangolin Medical, 2021) representing the target anatomy, being the left heart with inflow via pulmonary veins, and outflow via aorta, carotid, subclavian, left iliac, and femoral arteries. A 1/100 mix of dish soap and water is circulated through the phantom at constant flow using a centrifugal pump and a 15L buffer tank. According to cardiologists who tested the setup, this mix led to the most realistic friction between the instruments and artery walls in this model. At static pressure and flow, the liquid enters through the pulmonary veins into the left atrium, passes through the left ventricle and aorta, eventually exiting through the subclavian, carotid, brachiocephalic and left iliac arteries.

Similarly to a standard procedure in human, the instruments are inserted through the right iliac artery of the phantom. The device is used with the sheath fully inserted, the intraventricular guidewire is present in the left ventricle, and the tip of the delivery catheter at the sheath exit in the abdominal aorta. The robot is fixed to a static optical breadboard (*Newport, Irvine, CA USA*). Instruments can be mounted and unmounted at will. A webcam and floodlight are placed at a fixed position to provide an equivalent view on target anatomy for valve positioning, analogous to a standard TAVI fluoroscopy view. Two additional webcams are used for recording robot behavior (S1).

##### Phantom test bench for fluoroscopy

For the fluoroscopy experiments, the same pangolin phantom (Pangolin Medical, 2021) was used. A custom aortic valve phantom was placed in the annulus. The phantom was put under static pressure of the same liquid applied using a drip bag at a fixed height. A pigtail catheter is inserted through the brachiocephalic artery and nested in a valve cusp to mark its position in the fluoroscopic image.

##### *In vivo* setup

As *in-vivo* model, four domestic pigs (*Sus Scrofa Domesticus*) of breed large white were used, weight range 74.5 ± 7.5 kg. Procedures were acute and took place under general anesthesia, followed by euthanasia. All experiments were done in accordance with the ethics approval and guidelines of the research institute regarding the care of research animals, after the Committee of Ethical Animal agreement (APAFIS #38157), in accordance with the EU Directive 2010/63/EU for animal experiments. All invasive procedures were performed under general anesthesia and according to strict aseptic conditions. After intramuscular sedation with 20 mg/kg ketamine and 0.11 mg/kg acepromazine, the animals were placed in dorsal recumbent position. Intravenous access was obtained through a venous catheter inserted into a large atrial vein. Induction of anesthesia was obtained by 2 mg/kg propofol and maintained with 2% sevoflurane gas by mechanical ventilation (Dräger Zeus®, Dräger Inc, Telford, PA, US) and continuous 24 μg/kg/h fentanyl perfusion. At the end of the procedure, pigs were euthanized with 180 mg/kg pentobarbital.

Choice of animal was informed by the research center and based on anatomical similarity in terms of heart and vasculature as well as its prevalence and acceptance in cardiovascular *in-vivo* research. The experiments took place in the radiology room of the CERIMED research center in Marseille, France. The room was equipped with a OEC One mobile C-Arm (*GE Healthcare, Chicago, IL U.S.A.*). Field of view was limited when compared to more recently available imaging systems in modern hybrid ORs. The C-Arm was set up to maximize field of view and anatomy visibility as per review of a TAVI operator. The following features were prioritized: left ventricle, annulus, native valve, pigtail catheter distal end, safari guidewire distal end, delivery system nosecone, delivery system balloon markers and delivery system triple marker.

##### *In vivo* procedural technique

For each animal, two-point surgical access was performed. For the primary access, an incision was made in the abdominal space and the bowels were cleared to obtain visual access to the iliac arteries. Needle-based Seldinger technique was used to access the artery and standard TAVI steps were followed until the intraventricular guidewire was inserted inside the left ventricle and the large bore introducer sheath was in position. The secondary access was a percutaneous needle-based access in the carotid artery in order to place a pigtail catheter inside a native valve cusp.

In the cases where a valve was implanted, the standard delivery system and valve preparation steps were followed by a trained nurse and one operator. Geared rollers were assembled on top of the handle knobs to interface with the robot. An in-line pressure sensor and 1m length of high-pressure injection tubing are added between the inflation device and the delivery handle in order to enable pressure-monitored robotic balloon inflation.

The delivery system was then inserted through the large bore introducer sheath into the abdominal aorta. At this point, the robotic instrument driver was uncovered and moved in position. Large bore introducer sheath, delivery catheter, delivery system handle, guidewire, and inflation device are docked to their respective drivers using quick-release features. At this point, the technology can be used to robotically manipulate the instruments.

### Autonomy research

#### Computer vision on testbench

Seven videos of robotically navigating the instruments inside the phantom were recorded using different lighting and camera angles. A total of 668 frames were extracted from these videos and hand annotated to identify four features: the Left Ventricle (LV), the annulus reference (marked by a red line on the phantom annulus), the SAFARI^2^ guidewire coil and the yellow nosecone of the delivery system using the CVAT software (CVAT.ai Corporation, 2023). These frames were randomly split into 567 training frames and 101 validation frames. A yolov7 (Wang C et al., 2022) neural network was trained on the data for 100 epochs on a laptop computer with a RTX3060 GPU *(ROG Scar15, Asus, Taipei Taiwan).* Training hyperparameters are detailed in S5. This neural network allows us to robustly detect all four features in real time using 1,080 × 1920 image resolution at 25 frames per second.

**FIGURE 7:**
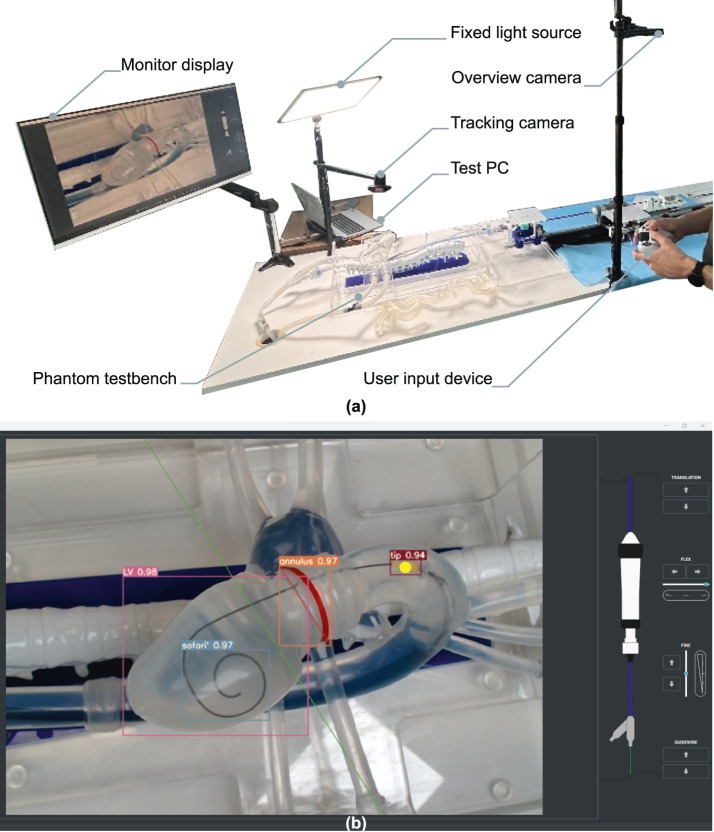
Illustrative example of operator experiment setup. **(a)** Situational overview example in lab conditions, camera vision phantom test setup. **(b)** The user interface as displayed on the monitor. Live-tracking overlays of the delivery system nosecone (tip), native annulus (annulus), left ventricle layout (LV) and intraventricular guidewire coil (safari) are shown. A fixed target depth is defined prior to the experiment as a live-tracked offset to the annular tracking, and visualized as a green line.

The detection derived from the neural network is then used to automatically position the delivery system. Using the box detection of the image, we can approximate the annular plane (in red on Figure 7). Using this line representing the annulus, we can place the target position for the nosecone at a tunable depth from the annulus (represented by a green line on Figure 7). The nosecone is rigidly fixed to the balloon, so controlling its position is a good proxy for controlling the balloon (and thus implant) depth. The nosecone position is represented by the center of its detection box (yellow dot in Figure 7). Once the target line and the position of the nosecone have been computed, the distance between the point and the line is computed. The use of camera vision on a translucent phantom provides an inherently safe, low-barrier entry point for robotics research without radiation safety considerations, which can then in turn be transferred to live use with fluoroscopy in an OR environment.

#### Computer vision with camera

Computer vision algorithm adaptation to fluoroscopy images on phantom was done using 405 images, acquired from three different sources. Data augmentation was applied to reach 1,262 images. CVAT (CVAT.ai Corporation, 2023) was used to annotate the pigtail coil, SAFARI coil, delivery system triple marker, balloon marker and tip. The data was randomly split into 1,125 training frames and 137 validation frames to train a yolov7 neural network as described previously.

Post processing was applied to the neural network output to determine the distance between the balloon marker and the annulus. First a Catmull-Rom spline interpolation was applied to the delivery system markers and the bottom right corner of the SAFARI bounding box. The local orthogonal vector to the spline passing through the pigtail center is then computed. This segment is used as a depth positioning reference, representing a proxy for the annular plane. Finally, we compute the distance between the center of the balloon marker and the depth positioning reference.

#### Closed loop controller

In both cases we obtain a distance to minimize as our control objective. Two independent PID controllers were designed and tuned to minimize the position error by actuating both the catheter body in translation and the fine adjust wheel respectively. The first PID is called the Macro positioner, it acts on the catheter translation when the error is larger than 20 pixels (approx. 6 mm). The second PID is the Micro controller, it acts on the fine adjustment wheel to finalize positioning when the error is smaller than 20 pixels.

Tuning was done manually and in function of expert operator feedback which indicated a preference for no overshoot. A critically damped response was targeted and reviewed in multiple instrument configurations. Parameters were identified and fixed separately for both camera vision and fluoroscopy feedback systems, with the primary variable being framerate between both systems.

### Operator review

#### Test method and protocol

The objective of this experiment was to evaluate the positioning performance of the robotic platform in two scenarios (teleoperated and autonomous positioning) compared to two manual positioning scenarios (with and without computer vision augmented imagery). The intention was to demonstrate at minimum equivalent positioning accuracy and precision of the technology with respect to that of an experienced TAVI operator on the phantom testbench. Furthermore, the aim was to review relative improvements of technological augmentations when compared to a manual case.

The operator was asked to stand between 1.5m and 2m from a screen displaying the user interface as in a typical hybrid OR. Control of the instruments was performed manually or via a gamepad controller, *(Dualshock4, Sony Interactive Entertainment, San Mateo, USA)*. The sequence of test cases was changed between operators to avoid learning effects across test cases. No clear trend in terms of performance change over time was observed, implying no occurrence of either learning effect or task fatigue.

Before starting the experiment, the operator was given 15 min to familiarize themselves with the task, robot, test bench and user interface. The task then consisted of navigating a delivery system balloon from a known starting position in the ascending aorta, to a planned delivery depth w. r.t the native annulus.

Four test cases were performed per experiment, each repeated 25 times: manual handling without visual augmentation, manual with visual augmentation, robotic teleoperated with visual augmentation and robotic with automated positioning. In each of the tasks the operator was asked to announce when they thought the final position was acceptable. This position was then recorded for 3 s before the experiment was reset.

#### Test cases

Four cases were reviewed:

• Manual actuation: the delivery system is manipulated manually and positioned without a reference for implantation depth. The annular tracking feature is provided as a visual reference target, to which the operator applies a relative visual estimate for a pre-defined target depth.
• Manual actuation with augmented vision: the delivery system is manipulated manually and positioned using camera feed with a feature tracking overlay. Pre-planned implantation depth is displayed and dynamically tracked w.r.t. the annulus.
• Teleoperative with augmented vision: the delivery system is actuated by the robotic system, controlled by a wireless user interface, using camera feed with a feature tracking overlay. Pre-planned implantation depth is displayed and dynamically tracked w.r.t. the annulus.
• Automated: the delivery system is actuated by the robotic system, controlled using a feedback control on position enabled by a dead man switch, using camera feed with a feature tracking overlay. Pre-planned implantation depth is displayed and dynamically tracked w.r.t. the annulus.

#### Data post processing

For each of the four cases, the orthogonal distance error between the center of the nosecone and the target line was measured and recorded. Final position error was defined as the average of a 3 s static measurement of the final position. These final errors are displayed as a boxplot. The median, first quartile and third quartile of the sampled data are displayed on the boxplots. Given the small number of samples, no outlier rejection was applied.

The pixel to millimeter ratio was measured to be 0.16mm/pix, locally calibrated in the region of interest of the camera frame using a linear scale. Sensor precision is determined as the 95% confidence interval across a >300s static measurement of the distance error with the instrument in the target region, and was measured to be 0.32 mm (2 pix). Camera field of view was set to mimic that of fluoroscopic interventional imaging, showing the aortic root, native valve, and left ventricle. Analogous to TAVI clinical practice, implantation depth error is reviewed as a 2D planar distance while minimizing parallax error via an orthogonal view to the native valve axis.

### Fluoroscopy autonomous positioning experiment

#### Test method and protocol

The objective of this experiment was to evaluate the positioning performance of the robotic platform in two scenarios: autonomous positioning with camera vision and autonomous positioning with fluoroscopy vision. The aim was to prove equivalence of performance in terms of accuracy and precision on both tasks.

The webcam experiment was repeated 30 times and the fluoroscopy experiment 31 times. The pixel to mm ratio and sensor precision are the same as the operator review as prior described. The pixel to mm ratio for the fluoroscopy task was determined to be 0.51mm/pix, with sensor baseline precision being a 1.4 pixels static noise band (corresponding to 0.71 mm). Given that pixel resolutions of the webcam and the fluoroscopy are different, all measurements were converted to millimeters in order to make them comparable.

## Data Availability

The original contributions presented in the study are included in the article/Supplementary Material, further inquiries can be directed to the corresponding author.
