# Supplementary material for: Towards autonomous robot-assisted transcatheter heart valve implantation: in vivo teleoperation and phantom validation of AI-guided positioning
Source: Front Robot AI. 2025 Oct 21;12:1650228. doi: 10.3389/frobt.2025.1650228 (PMC12583050; doi:10.3389/frobt.2025.1650228)
Supplement: Supplementary file 2 [file Supplementaryfile1.pdf]

Robotic device architecture - autonomy

System Description

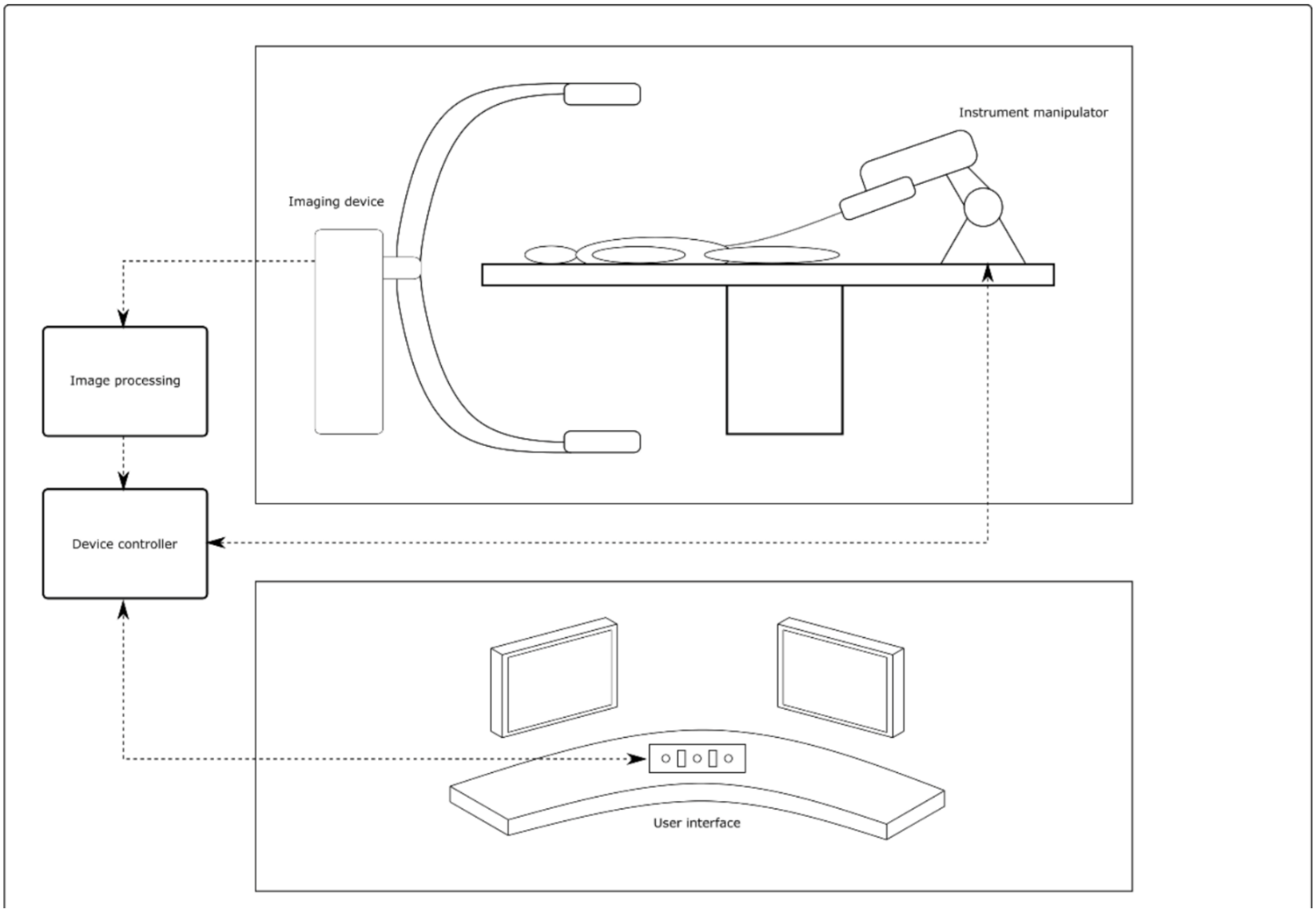

The above figure shows a conceptual description of the overall system for closed loop position control. This section will provide a definition as well as a description of each subsystem.

Interventional actions are performed using a robotic **instrument manipulator**, which interfaces with the surgical instruments used to position and deliver an aortic THV implant. The device is fixated to the operating table, maintaining a fixed position w.r.t. the patient anatomy. All required instrument functionalities are enabled via actuators. It should be noted that the instruments remain convertible to manual at all times.
An **imaging device** provides intraoperative imaging data which, at minimum, enables to infer the relative position of a transcatheter valve w.r.t. a native valve anatomy target. For instance, a fluoroscopy device. Alternatively, in some phantom experiments described in this article, a camera was used by proxy.

In turn, an **image processing unit** receives the image or video feed from the imaging device, and processes it in order to output meaningful variables for determining interventional actions. At minimum, the image processing unit infers the implant and the target implantation depth and computes the depth difference as an output.

A **user interface** is provided to the operator and consists of two parts: 1) a monitor that shows the intraoperative imaging with meaningful overlays as well as a status screen providing information

on functioning of the overall device. 2) a physical input device allowing the operator to instruct the system to perform interventional actions.

The instrument manipulator, user interface, and image processing unit all interface with the **device controller**, which manages the overall system state and its behavior. In its simplest form, the device controller manages the initialization of the device, translates user inputs to tele-operated instrument actions, and enables closed-loop positioning control behavior on operator-command.

What follows is a more detailed description of the robotic instrument manipulator, inflation driver, and user interface.

PROTOTYPE DESCRIPTION – feasibility demonstrator, non-clinical R&D, idea to in-vivo
The goal of this technology phase is demonstrating feasibility of robotic TAVI in a real-world environment. A primary initial design constraint is the preservation of the commonplace TAVI workflow, preservation of 3rd party surgical instruments, and the ability to convert to a commonplace procedure at any given point without loss of function or procedural impact.

A principle objective was enabling full robotic control of all gestures related to implant positioning and deployment during TAVI. A solution architecture is proposed, following a functional analysis of real-world TAVI workflows and expert input. Implementation is done for a specific balloon-expandable TAVI platform (Edwards Lifesciences, SAPIEN3), yet the architecture remains applicable to other balloon- or self-expanding TAVI platforms with minor change of function.

The design philosophy is one of rapid prototyping, prioritizing speed and relevant adaptability while delivering a sufficiently high level of robustness to reliably run tests in real-world conditions. Functional decomposition allows for stand-alone iterations, minimizing system-wide impact of pivots. Most of the design is enabled by standard off-the-shelf mechanical components, combined with in-house 3D printing. Versatile off-the-shelf servo actuators are initially chosen with a reasonable performance margin to allow for ad-hoc adjustment or concept pivots.

Architecture and concept selection is done with safe and effective clinical end-use in mind, but it is readily understood that this is not exhaustively addressed during feasibility phase. Following non-clinical feasibility demonstration, the solution architecture undergoes a major redesign to enable clinical investigational use, driven by medical device design controls, including risk management, human factors, and compliance to relevant harmonized standards. Such is out of scope for this report. Future work includes a clinical review of a next generation technology.

Instrument Manipulator – Architecture & functional layout

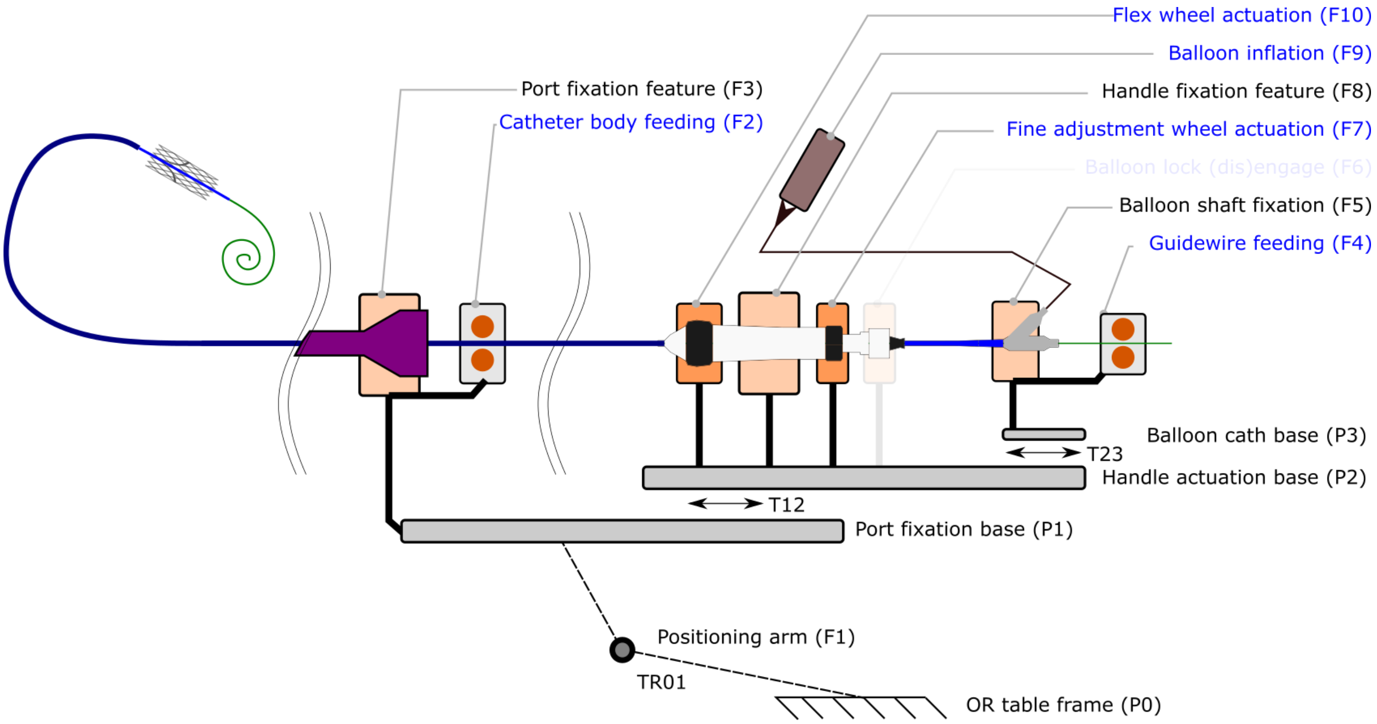

Catheter Driver

The catheter driver realizes two features: it fixates the proximal end of the introducer sheath and it actuates the delivery catheter in translation.

The introducer sheath fixation feature is realized by a custom fixture held shut and tensioned with a thumb-screw.

To realize the delivery catheter translation feature, the catheter driver is comprised of 2 pairs of rollers, 1 driving pair and 1 centering pair. The 1 driving roller pair is driven by 1 servomotor (Dynamixel XM430-W210-T) through 40:24 belt transmission. One driving roller is fixated on linear guides, enabling a translation opposite of the static row of rollers. The centering roller is also mounted on a separate linear guide and spring loaded in a closed configuration. This allows to insert or remove the delivery catheter from above. The gripping force of the driving pair is managed through a single screw, tightened using a hex key. Covers were added to protect the actuators and mechanical components, as well as for improved cleanability and aesthetics.

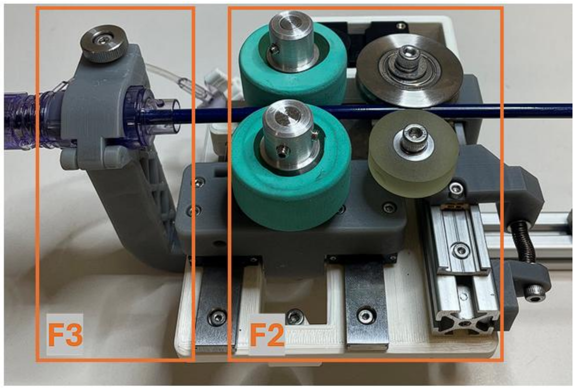

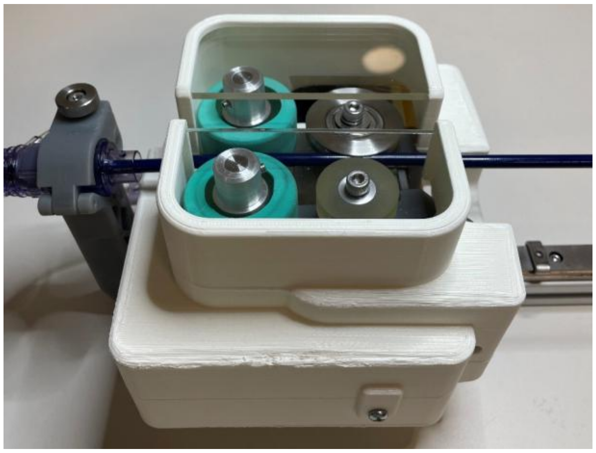

Handle driver

The handle driver realizes three features: it fixes the delivery system handle, and it actuates both knobs on the handle. The handle fixation feature is realized by a custom fixture held shut and tensioned with a thumb-screw.

To interface with and actuate both knobs, custom interface parts were designed. These match the grooves of the knobs on the inside and are geared on the outside. These interface parts are in turn actuated by a gear rigidly attached to a servomotor (Dynamixel XM430-W210-T). Gear interfaces 50:40 and 40:40 for the Flex Knob and the Fine Adjustment Knob respectively.

The whole handle driver mounted on a passive linear rail (DOLD Mechatronik). As such, the delivery catheter is able to freely translate w.r.t. the patient frame and remains manually overridable at all times.

Covers were also added to protect the actuators and mechanical parts, as well as for improved cleanability and aesthetics.

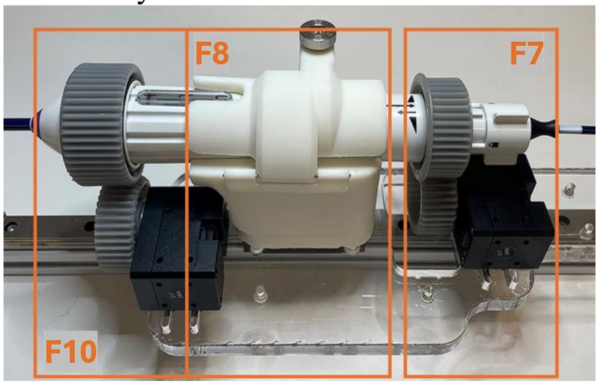

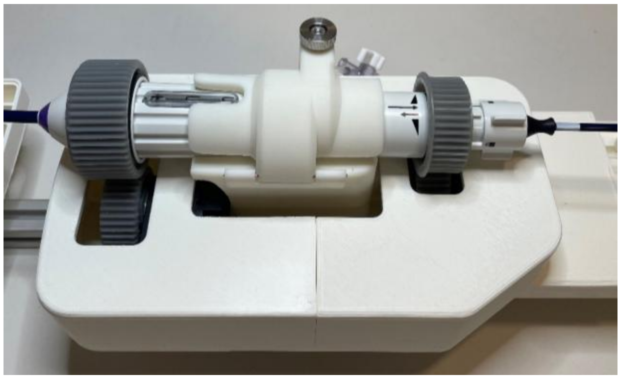

Guidewire Driver

The guidewire driver realizes two features: it fixes the Y-connector at the proximal end of the delivery system, and it actuates the guidewire in translation.

The Y-connector fixation feature is realized by a custom fixture held shut and tensioned with a thumb-screw.

To realize the guidewire translation feature, the guidewire driver is comprised of 2 pairs of rollers, 1 driving pair and 1 centering pair. The 1 driving roller pair is driven by 1 servomotor (Dynamixel XM430-W210-T) through 40:24 belt transmission. One driving roller is fixated on linear guides, enabling a translation opposite of the static row of rollers. The centering roller is also mounted on a separate linear guide and spring loaded in a closed configuration. This allows to insert or remove the guidewire from above. The gripping force of the driving pair is managed through a single screw, tightened using a hex key. An additional centering feature is added to the back of the guidewire driver, consisting of a funnel like channel positioned on the axis of the guidewire. The distal centering of the wire is managed by the Y-connector on the delivery system.

The whole guidewire driver is mounted on a passive linear rail (Misumi). As such, the balloon catheter is able to freely translate w.r.t. the delivery system handle and remains manually overridable at all times.

Covers were also added to protect the actuators and mechanical parts, as well as for improved aesthetics.

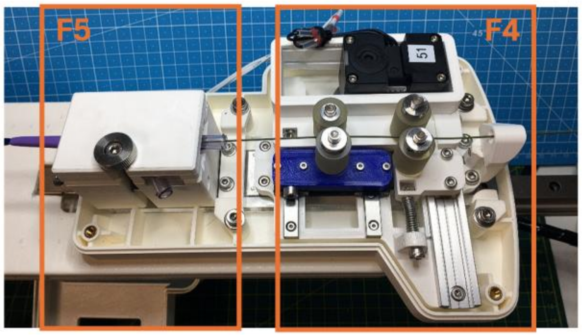

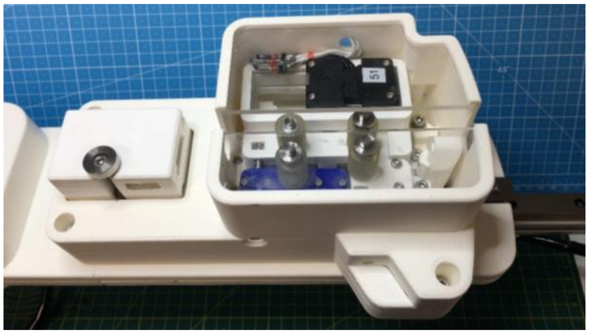

Inflation Driver

An inflation driver was developed to enable robotic control of Edwards balloon inflation, not requiring the operator to directly manipulate the inflation device.

The driving mechanism is realized by a linear spindle drive (FUYU Technology) driven by a NEMA23 stepper motor. The drive carriage is connected to the syringe plunger, while the syringe housing is fixated to the drive base. This allows to robotically advance or retract the syringe plunger. A L-shaped custom bracket and adapter were designed allow for easy assembly of the syringe in the inflation driver and allows to quickly convert back to a manual procedure if necessary. A slide potentiometer is used to track the position of the plunger in the syringe barrel and an in-line pressure sensor is used to monitor the balloon pressure during use.

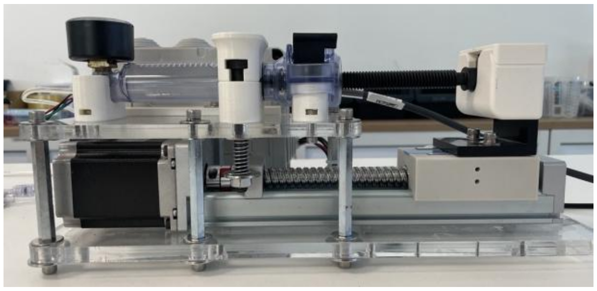

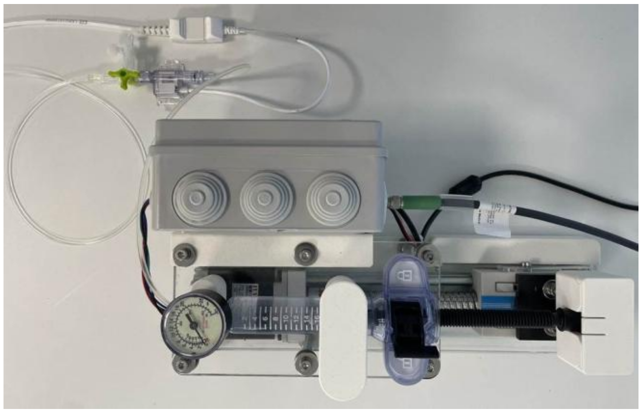

Device control
Low level control and signal processing is done using a custom PCB assembly, with embedded software running on an off-the-shelf microcontroller (Teensy 4.1). High level device control, inference, logging, and visualization are done using a standard laptop PC.

User Interface
Visual feedback is provided to the user via an "Operator Panel", shown on a monitor display. This panel provides a view of the fluoroscopy feedback as well as information on the robot actuation, represented by buttons which light up when features are being used, as can be seen on the figure below.

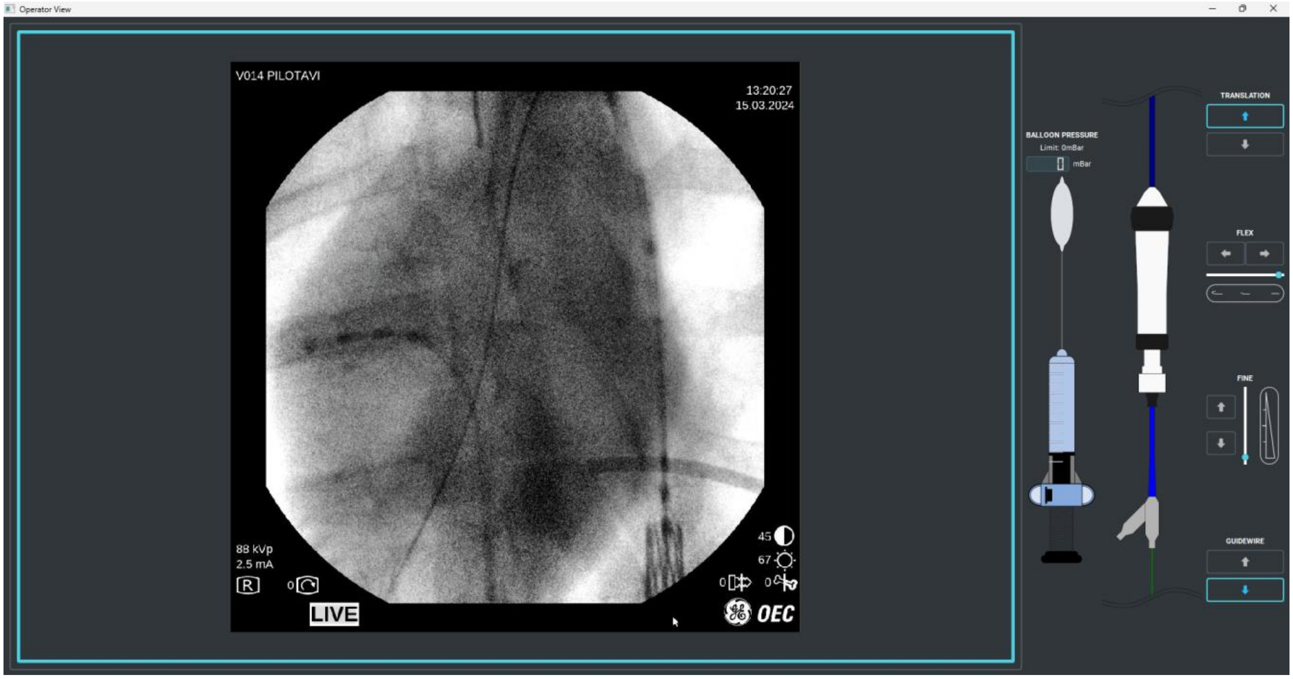

An "Engineering UI" was also developed and can be seen in some of the supplementary videos. It provides the user with detailed information on metrics from the motors (position, speed…), as well as control over some variables (limits to speeds and pressures).

In order to control the system, the operator uses a consumer-grade gamepad (Sony PlayStation Dualshock 4). On this gamepad are mapped all of the low level motion features (translation and rotation of instruments and knobs), high level motion features (autonomous motion controller, synchronous mode) and some initialization and debugging features (Range calibrations, motor reset). Alternatively, the engineering UI can also be used to control the robot via a mouse and keyboard interface.

Neural Network hyperparameters

lr0	0.01
lrf	0.1
momentum	0.937
weight_decay	0.0005
warmup_epochs	3.0
warmup_momentum	0.8
warmup_bias_lr	0.1
box	0.05
cls	0.3
cls_pw	1.0
obj	0.7
obj_pw	1.0
iou_t	0.2
anchor_t	4.0
fl_gamma	0.0
hsv_h	0.015
hsv_s	0.7
hsv_v	0.4
degrees	0.0
translate	0.2
scale	0.5
shear	0.0
perspective	0.0
flipud	0.0
fliplr	0.5
mosaic	1.0
mixup	0.0
copy_paste	0.0
paste_in	0.0
loss_ota	1
batch size	8
